# P-1430. Does BCG Vaccine Result in Non-Specific Effects on Childhood Infections in American Indian and Alaska Native Children?

**DOI:** 10.1093/ofid/ofaf695.1617

**Published:** 2026-01-11

**Authors:** Andrew W Hill, Cara Olsen, Martin Ottolini, Naomi E Aronson, Joy Abraham

**Affiliations:** Walter Reed National Military Medical Center, Chevy Chase, MD; Uniformed Services University, Bethesda, Maryland; Uniformed Services University of the Health Sciences, Bethesda, Maryland; Uniformed Services University of the Health Sciences, Bethesda, Maryland; 81st Medical Group Keesler Air Force Base, Biloxi, Mississippi

## Abstract

**Background:**

Non-specific effects (NSE) of vaccines such as BCG may protect against subsequent infections beyond their target pathogens, via both innate and adaptive immune mechanisms. We hypothesized that BCG vaccination would be associated with lower rates of common pediatric infections.

**Methods:**

We performed a secondary analysis of data collected during a placebo-controlled trial of BCG vaccination among American Indian/Alaska Native (AI/AN) schoolchildren, conducted from 1935–1938. This study was approved by the USUHS Institutional Review Board. The trial enrolled 2,860 AI/AN schoolchildren and prospectively recorded post-vaccination medical histories through 1947. Inclusion criteria were negative tuberculin skin test, normal chest radiograph, and receipt of study vaccine. Children with prior varicella, parotitis, measles, or pertussis were excluded. Outcomes included subsequent pneumonia/pleurisy, pertussis, measles, mumps, rubella, varicella, influenza, and infectious (non-TB) mortality. Cox proportional hazards models estimated hazard ratios (HRs) and 95% confidence intervals (CIs), adjusted for age, sex, and region. Kaplan-Meier time-to-event analyses were also performed.

**Results:**

Among enrolled children, 1,489 received BCG and 1,371 placebo. Adjusted HRs (95% CI) were: non-TB infectious mortality 0.79 (0.54–1.47), pneumonia 0.84 (0.64–1.11), rubella 0.94 (0.64–1.37), varicella 0.95 (0.70–1.29), mumps 0.98 (0.80–1.19), influenza 1.01 (0.80–1.28), measles 1.06 (0.88–1.27), and pertussis 1.08 (0.85–1.37). Log-rank tests suggested differences between BCG and placebo for pneumonia (p=0.21), pertussis (p=0.57), measles (p=0.52), varicella (p=0.74), rubella (p=0.71), mumps (p=0.79), and influenza (p=0.92).

**Conclusion:**

Five of eight outcomes had HRs < 1.0, suggesting a trend towards protective effects. However, none reached statistical significance. BCG vaccination in school-aged children trended toward NSE protection but did not significantly reduce infection rates or infectious mortality in this pre-vaccine era population.

Disclaimer: The views expressed in this abstract are those of the author(s) and do not necessarily reflect the official policy of the Department of Defense or the US government. Authors report no conflicts of interest.

**Disclosures:**

Naomi E. Aronson, MD, Wolters Kluwer: royalties for writing for uptodate

